# Sulforaphane alter the microbiota and mitigate colitis severity on mice ulcerative colitis induced by DSS

**DOI:** 10.1186/s13568-020-01053-z

**Published:** 2020-07-03

**Authors:** Yan Zhang, Luxuan Tan, Chao Li, Han Wu, Dan Ran, Zhenyu Zhang

**Affiliations:** 1 Department of Gastroenterology, Nanjing First Hospital, Nanjing Medical University, 68 Changle Road, Nanjing, 210006 China; 2 Department of Gastroenterology, The Second Hospital of Nanjing, Nanjing University of Chineses Medicine, Nanjing, 210003 China

**Keywords:** Sulforaphane, Gut dysbiosis, Ulcerative colitis

## Abstract

Sulforaphane (SFN) is a kind of natural isothiocyanate, which exists in cruciferous plants. Only few studies were about the anti-inflammatory effects of sulforaphane in ulcerative colitis. In this study, our purpose is to explore the effects of sulforaphane on the intestinal microbial community of UC mice. The severity of mice colitis were measured by colon length, survial rate, body weight and disease activity index (DAI) score. Histological and morphological evaluation of colon tissues were performed by HE. 16S rRNA gene amplicon pyrosequencing was used to analyza the changes of mouse flora. The variety of flora expression were explored using quantitative PCR. Sulforaphane treated mice had larger body weight and longer colon length than DSS-induced mice. The colon tissues of DSS group showed congestion and edema. Meanwhile, treatment with sulforaphane effectively reducted the damage scores and MPO activity. Sulforaphane reversed DSS-induced gut dysbiosis. Sulforaphane would shift the balance to *Butyricicoccus* on inflammation. The possible anti-inflammatory mechanism of sulforaphane is to coordinate with the probiotics such as *Butyricicoccus*. In summary, these findings proved that sulforaphane might be a useful content and serve as a potential therapy in the treatment of UC.

## Introduction

Ulcerative colitis (UC) is a kind of chronic nonspecific inflammatory bowel disease. Epidemiological studies shown that the prevalence of UC in the world is 5.50–24.30 in 100,000. In North America and Europe, the prevalence of UC is higher, about 24.30 in 100,000 and 19.20 in 100,000 respectively. In Asia and the Middle East, about 6.30 in 100,000 (Ng et al. 2013). Until now, the etiology of UC is unclear. It can be related to heredity, geographical environment, dietary habits, autoimmunity, smoking or other factors.

Recently, more and more studies have shown that intestinal flora has a high correlation to the occurrence and development of UC. Many factors, espicially diet and environment, can react to intestinal flora and then causes flora imbalance (Guinane and Cotter 2013; Goto et al. 2015). Normally, intestinal flora would not cause colonic mucosal inflammation directly. When the host is in a stressful environment or some pathological conditions, harmful bacteria or opportunistic pathogens in the intestine have a higher-risk to colonize, invade the intestinal epithelium and cause inflammation (Yang et al. 2017). However, the causal relationship between intestinal flora imbalance and UC remains controversial (Pei et al. 2019).

Sulforaphane, the main hydrolytic active product of glucosinolates, belongs to the isothiocyanate family and widely exists in cruciferous vegetables such as broccoli, kale and Northern carrot. Broccoli, especially 3-day-old broccoli buds, contains a high concentration of sulforaphane, which is the main source of substances in our clinical research (Liu et al. 2008). Wagner et al. (2013) established an animal model of DSS-induced UC which is pretreated with SFN at a dose of 25 mg/kg. The results showed that SFN could alleviate diarrhea, weight loss and colon tissue damage in mice, and reduced inflammatory biomarkers such as interleukin-6 and interferon-gamma. In mdr1a (−/−) IBD mice, SFN-rich fresh broccoli diet has been shown to alter intestinal microflora and alleviate colitis (Paturi et al. 2012).

However, the effect of sulforaphane on intestinal flora is unclear. Therefore, a DSS-induced UC mice model used to study the intervention of SFN on UC and observe the effect of SFN on intestinal flora, in order to provide new ideas for the treatment of ulcerative colitis.

## Materials and methods

### Animals and grouping

18 healthy male C57BL/6 (18–22 g) were purchased from the laboratory animal center of Nanjing medical university. Randomly divided animals into three equal groups, including the dextran sulfate sodium induced group (called DSS group below), SFN group, and blank group. In 2 weeks, SFN group was given SFN intragastric administration 20 mg/kg/days, other two groups were given distilled normal saline. But start from the second week, SFN group and DSS group were administrated 2.5% DSS in drinking water to induce UC additionly. Body weight was measured daily at the same time. At the end of the experient, sacrifice the mice by cervical dislocation and then collect the colon specimens. l-Sulforaphane was gift from Huahan Biotechnology Co.Ltd, Ganzhou, Jiangxi, China (batch number: 20180101).

### Histological and morphological evaluation

Colon tissues were preserved in 10% neutral formalin. Then embedded the tissues in paraffin and stained with hematoxylin and eosin (HE). A standard histological scoring system was used as a criterion to evaluate colitis. Briefly, 0 = no damage; 1 = extent of disease < 25%; 2 = extent of disease 26–50%, 3 = extent of disease 51–75%, 4 = extent of disease > 75%.

### UC severity evaluation

UC severity evaluation was based on the disease activity index (DAI) score which has been widely used in animal models. The researcher recorded and scored the changes following the protocol, which contained hemoccult positivity, weight, stool consistency and gross bleeding. Then all these parameters combined to DAI score for evaluation.

## 16S rRNA gene amplicon pyrosequencing

Collected fecal samples and extracted DNA with using the Stool DNA Kit (Omega Bio-tek, Norcross, GA, U.S) according to the manufacturer protocols. Using spectrophotometer and 1% agarose gel electrophoresis to measure the purity and concentration of microbial DNA. Next step was the V4–V5 region of the bacteria 16S rRNA gene amplicon, inwhere two universal primers 338F (ACTAATACGGAGGCAGCAG) and 806R (GGACTACNNGGGTATCTAAT) was used by PCR. The first step of PCR was 94 °C for 5 min, then did 28 cycles of 94 °C for 30 s, 55 °C for 30 s and 72 °C for 60 s, the final step was 72 °C for 7 min. Each PCR reaction system was consisted of 2× Taq Plus Master Mix (12.5 μL), BSA (3 μL with 2 ng/μl density), 1.0 μL of each primer (5 μM), ddH_2_O and 30 ng of template DNA in a 25 μl volume. PCR products were collected and purified with QIAquick Gel Extraction Kit (QIAGEN, Germany).

Pooled purified amplicons at equimolar ratios, then paired-end sequenced (2 × 250) on an Illumina MiSeq platform. The raw data were stored in the NCBI Sequence Read Archive (SRA) database. Using Trimmomatic to demultiplex and qualityfilter the raw fast files, and using silva to cluster Operational Taxonomic Units (OTUs) with 97% similarity cutoff. Then UCHIME was used to identify and remove chimeric sequences. The silva (SSU115) 16S rRNA database was analysed by RDP Classifier (http://rdp.cme.msu.edu/) with a confidence threshold of 70%. At each taxonomical level (Phylum, Class, Order, Family, and Genus), one analysis was performed respectively. The diversity of alpha (within samples) and beta (among samples) were analyzed by using Inhouse Perl scripts. The α diversity index was calculated by using Mothur software (version 1.31.2). The R (v3.1.1) software package is used for clustering analysis. UniFrac algorithm uses the information of system evolution to compare the differences of bacteria groups among samples, and makes further statistical analysis of the results.

### Quantitative PCR of butyricicoccus in fecal DNA of mice

Using Trizol (Invitrogen, USA) to extract the total RNA in colon tissue, and using RNA reverse transcription kits to reverse-transcrib RNA into cDNA. Upstream primer 5′-ACCTGAAGAATAAGCTCC-3′, Downstream primer 5′-GATAACGCTTGCTCCCTACGT-3′. Using a SYBR Premix EX Taq Realtime PCR Master Mix (TaKaRa) to check mRNA expression levels on a Bio-Rad Q5 instrument (Bio-Rad, CA, USA).

### Statistical analysis

Data collected from all three groups were represented as the mean ± standard error of the mean (SEM). More than two groups’ datasets were evaluated by one-way ANOVA followed by Newman–Keuls post hoc tests. Among all three groups, statistical significance of nonparametric variables’ differences was assessed by the nonparametric Turkey test followed by the Mann–Whitney U test when P < 0.05. The statistical analysis were performed by SPSS 22.0 software (Chicago, IL, USA).

## Results

### Effects of sulforaphane on UC clinical symptoms in DSS-induced mice

**Fig. 1:**
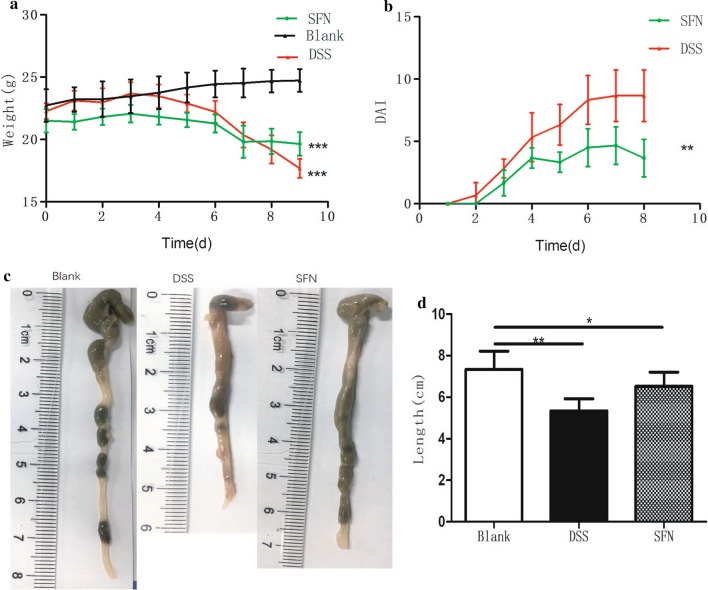
Effects of Sulforaphane (SFN) on clinical signs in DSS-induced colonitis. (n = 6 per group). **a** DSS-induced ulcerative colitis caused the loss of body weight in mice. **b** Disease activity index score in the SFN and DSS groups. **c**, **d** Effect of SFN on DSS-induced colon shortening. *P < 0.05, **P < 0.01, ***P < 0.001

Mice in the blank group had normal diet, activity, stool consistency, hair grown and weight gain. However, in the DSS group, the mice showed a obvious decrease in body weight and colon length (Fig. 1a, c, d). Moreover, as expected, the DAI scores of DSS-induced mice increased significantly (Fig. 1b). These mice showed anorexia, reduced activity, loose stool, drab hair color and weight loss.

Compared to the blank group, DSS-induced mice had obvious weight loss and lower DAI scores. The symptoms in the SFN group were milder than the DSS group.

### Histological observation and evaluation

**Fig. 2:**
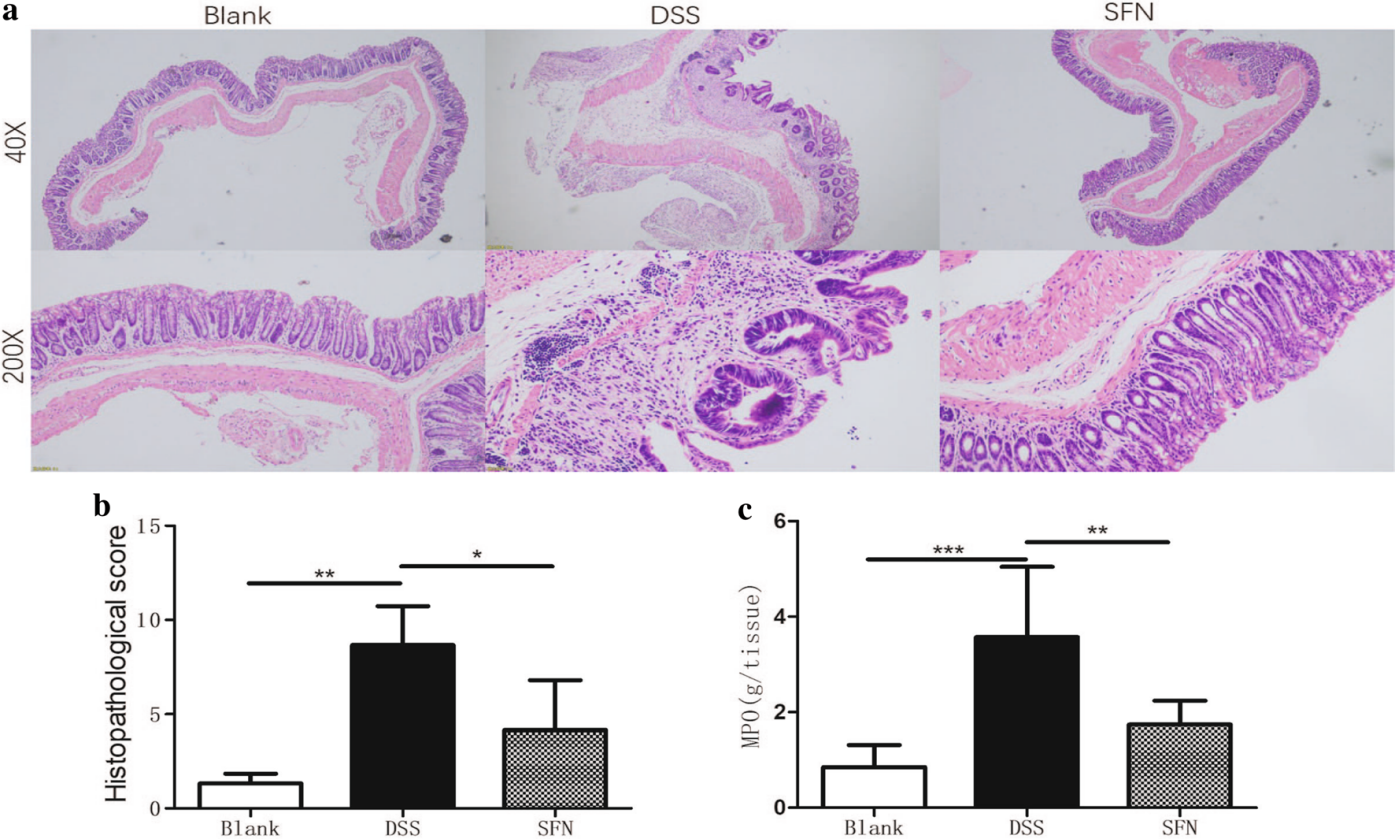
Sulforaphane (SFN) pretreatment alleviated DSS-induced colon inflammation (**a**) Histopathological changes after DSS stimulation in colon (×40, ×200). **b** Effects of SFN on microscopic damage scores. **c** Effects of SFN on MPO activity. *P < 0.05, **P < 0.01, ***P < 0.001

The estimation of damage extent of colon tissues was showed in histological analysis. HE staining of blank group showed normal colonic mucosal epithelium (Fig. 2a). Instead, DSS group had extensive glandular destruction and inflammatory cell infiltration in the submucosa. Compared with DSS group, SFN group showed milder degree of congestion and edema. As shown in Fig. 2b, c, SFN group and DSS group had a signifcant difference between the microscopic damage scores and MPO activity (P < 0.05). Moreover, the difference between blank group and DSS group was more statistically significant (P < 0.01).

### Sulforaphane reversed DSS-induced gut dysbiosis

**Fig. 3:**
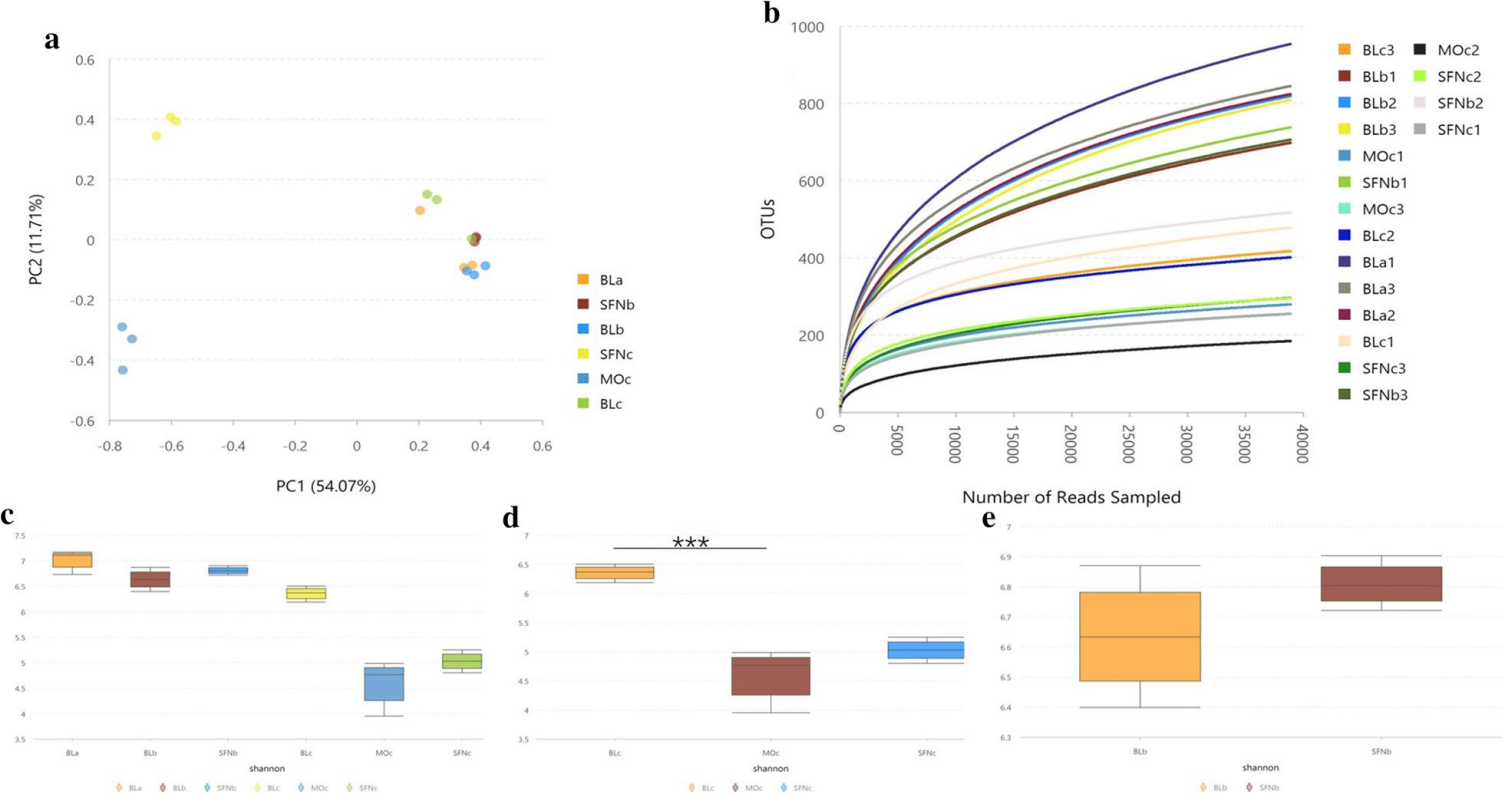
Sulforaphane (SFN) modulated the structure and diversity of gut microbiota. **a** Principal coordinate analysis (PCoA) and **b** Alpha diversity analysis at the OTU level of mouse’s fecal microbiota. **c**–**e** Shannon index in different group. Significant differences between blank vs. DSS are indicated: ***P < 0.001. Bl represents the blank group, SFN represents the SFN group, MO represents the DSS group. a represents day 0 of the group, b represents day 7 of the group, c represents day 14 of the group. As Moc represents the day 14 of the DSS group

For all the three groups, using Illumina HiSeq/MiSeq platforms sequenced variable regions V4–V5 of the 16S rRNA gene from fecal samples. The purpose was to evaluate the changes in the gut microbial community. Operational taxonomic units (OTUs) was uesd to present the results with a 97% homology cutoff value (Fig. 3). An obvious clustering of the microbiota composition was presented by principal coordinates analysis (PCoA) for all three group (Fig. 3a). The clustering of microbiota composition from PCoA and microbial communities from alpha diversity analysis at the OTU level in DSS group were significantly different from the other two groups. Instead, the SFN group and the blank group had a similar microbial community in the feces (Fig. 3b). The diversity was shown in Shannon index analysis (Fig. 3c–e). The higher value indicated the higher diversity richness. The community richness in the DSS group were lowe than other two groups (Fig. 3d, P < 0.001).

**Fig. 4:**
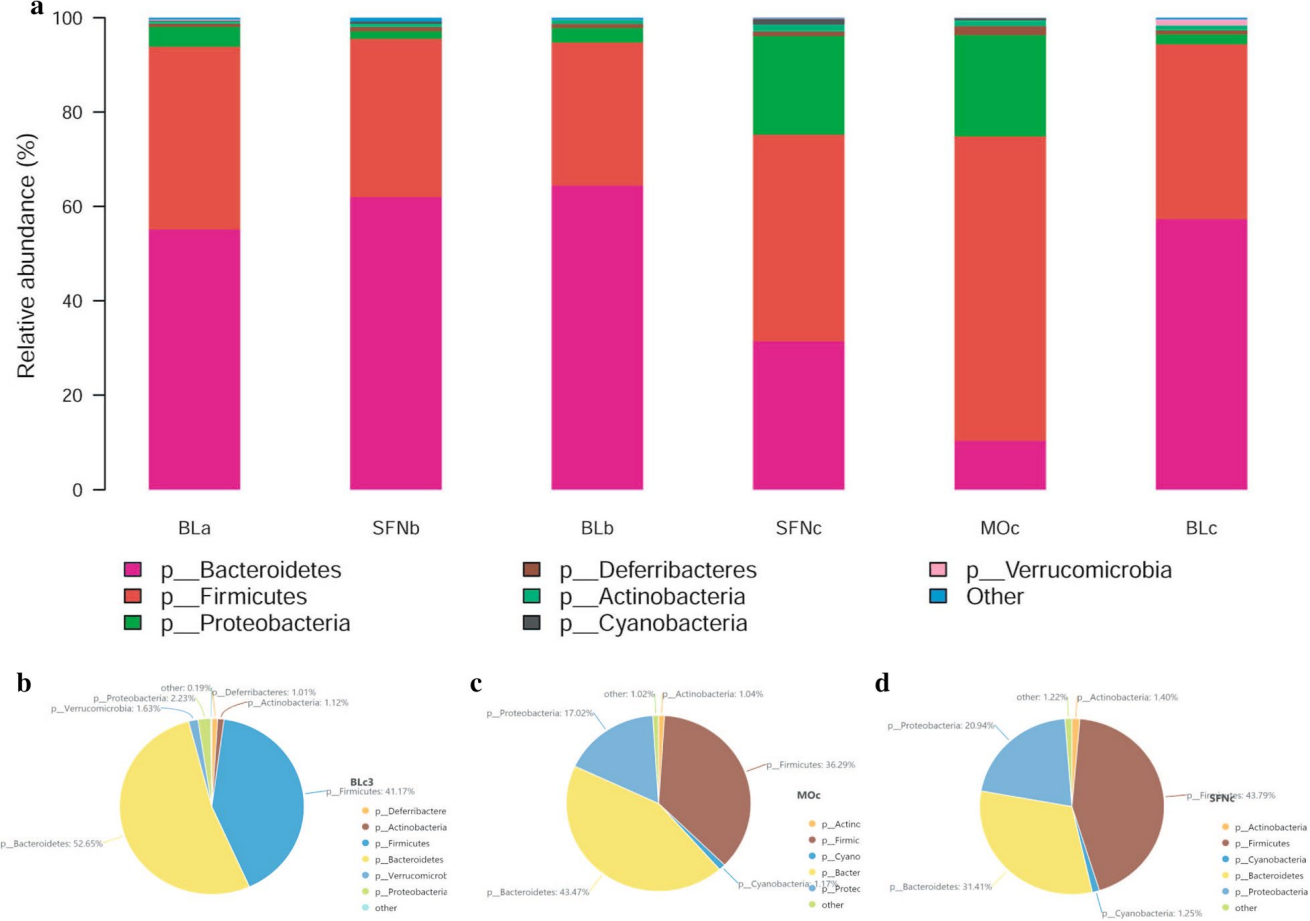
SFN modulated the composition of gut microbiota at the phylum level. **a** Phylum-level taxonomic distributions of the microbial communities in feces. **b**–**d** Composition of the phylum level in the 14th day in three groups

At the phylum level, the relative abundance of the predominant taxa identified from sequencing of all three groups were shown in Fig. 4, which can present the specific changes in the gut microbiota. Figure 4a illustrated a detailed overview of the intestinal bacteria composition. In this study, *Firmicutes*, *Bacteroidetes*, and *Proteobacteria* had a higher richness in all samples. Moreover, the three phyla differed wildly in each group. After 7 days, the intestinal bacteria composition of the SFN group had no statistic difference with other two groups. After 14 days, the relative abundances of *Firmicutes* in the DSS group was higher (Fig. 4c, P < 0.05), but which in the SFN group was lower (Fig. 4d, P < 0.05). The abundance of *Bacteroidetes* in the DSS group was the lowest, but SFN significantly increased the level of *Bacteroidetes*(Fig. 4b, P < 0.05). *Firmicutes*-*to*-*Bacteroidetes* ratio was a reliable indicator used herein to assess the gut microbiota. The *F*/*B* ratio in the blank group was 0.6469, and other two groups had an increased ratio. Moreover, the ratio showed siginificant difference between the DSS group and the blank group (P < 0.05).

At the family level, in the first 7 days, the blank group significantly increased the level of *Bacteroidaceae*, *Bacteroidales_S24*-*7*, *Lachnospiraceae*, *Ruminococcaceae*, *Rikenellaceae* and *Prevotellaceae*, and the SFN group had a similar trend. In the second 7 days, the abundances of *Lachnospiraceae*, *Bacteroidales_S24*-*7*, *Rikenellaceae*, *Lactobacillales* and *Prevotellaceae* was significantly decreased in the DSS group than in the blank group (P < 0.05). In contrast, the abundances of *Clostridiaceae_1*, *Erysipelotrichaceae*, *Campylobacteraceae*, *Peptostreptococcaceae* and *Enterobacteriaceae* increased in the DSS group than in the blank group. The SFN had an obvious inhibitory action to the relative abundance of *Bacteroidaceae*, *Enterobacteriaceae*, *Bacteroidales_S24*-*7*, *Rikenellaceae* and *Prevotellaceae*. Overall, these results indicated that SFN could prevent the gut dysibosis induced by DSS in mice.

### Sulforaphane would shift the balance to *Butyricicoccus* on inflammation

**Fig. 5:**
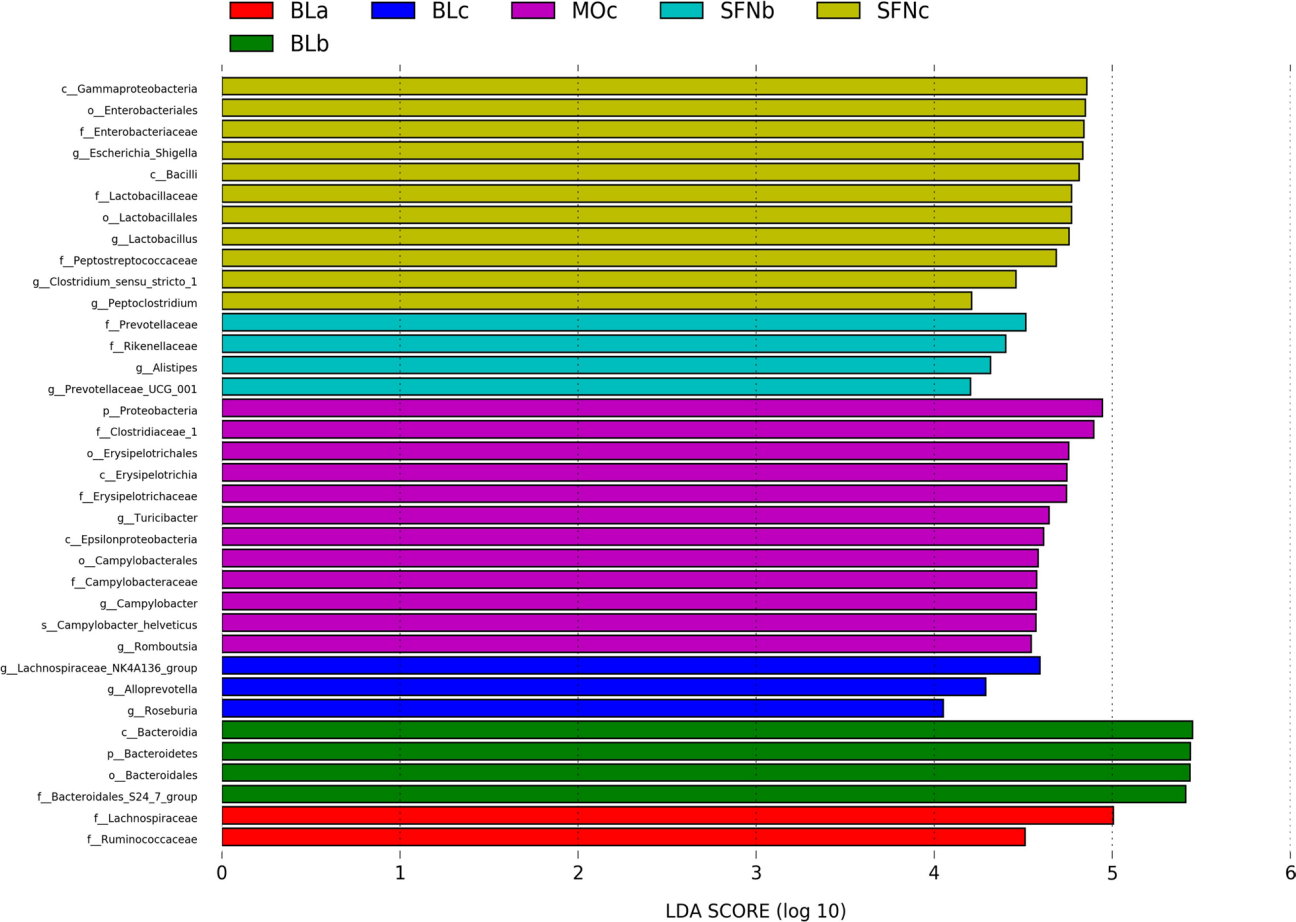
Comparison of species with significant difference in abundance among the three groups of mice by lefse analysis

In a further step, to identify the faecal microbiota meaningful changes among the three groups, the relative abundance of 36 genera were presented by branch diagram through Lefse analysis (Fig. 5). Obviously, different groups showed different genera levels. *Alloprevotella* belongs to *Bacteroidea*, *Lachnospiraceae NA4A136 group* and *Roseburia* belong to *Firmicutes*, which are the specific bacteria in the blank group. In the DSS group, *Epsilonproteobacteria*, *Campylobacterales*, *Campylobacteraceae*, *Campylobacter* and *Campylobacter helveticus* belong to *Proteobacteria*, *Erysipelotrichia*, *Erysipelotrichaceae* and *Turicibacter* belong to *Firmicutes*. Moreover, in the SFN group, *Gammaproteobacteria*, *Enterobacteriales*, *Enterobacteriaceae*, *Escherichia Shigella* belong to *Proteobacteria*, while *Bacilli*, *Lactobacillaceae*, *Peptostreptococcaceae*, *Clostridium* sensu stricto *1* belong to *Firmicutes*.

In order to further analyze the specific bacteria at the genus level, the metastats method was used to compare the bacteria between the DSS group and the blank group, and seven bacteria with statistical difference were selected (P < 0.01). In the blank group, the relative abundances of *Ruminiclostridium6*, *Alloprevotella*, *Butyricicoccus*, *Lachnospiraceae_UCG*-*006* and *Family_XIII_UCG*-*001* were increased. On the contrary, the relative abundance of *Turicabaracter* was reduced. Compared with the DSS group, the relative abundance of *Butyricicoccus* in the SFN group increased (P = 0.0337), while that of *Turibaccharacter* decreased (P = 0.0056).

To further study, the quantity of *Butyricicoccus* gene was detected by fluorescence. The quantity of *Butyricococcus* in the DSS group is much lower than the quantity in the blank group (Table 1, P < 0.05). The quantity of *Butyricicoccus* in SFN group was higher than that of the DSS group, but less than that of the blank group. SFN could reverse the changes of *Butyricicoccus* to the DSS group at the genus level.

**Table 1:** Quantitative analysis results of *Butyricicoccus*

Sample name	Copies	Copies	Copies	Average copies
SFNc	4.8709	10.7772	40.1199	18.5893
MOc	12.0912	1.5708	5.5293	6.4171
BLc	2847.3337	66.2252	72.8111	995.4567

## Discussion

Ulcerative colitis is one common chronic diseases of digestive system. At present in clinical, doctors use anti-inflammatory drugs or immunosuppressive drugs to treat UC. However, these drugs usually cause some side effects. UC has a long course of disease, which is easy to recur, and cannot maintain long-term clinical remission. The need of new treatment methods is urgent.

SFN is a kind of natural isothiocyanate, which exists in cruciferous plants, such as cabbage and broccoli. SFN can activate the nuclear factor E2 related factor 2 (Nrf2) pathway, and induce the body to produce type II detoxification enzymes, which have antioxidant, anti-cancer activity and immune regulation functions (Sun et al. 2015). In recent years, SFN has also been found to have good anti-inflammatory and antibacterial effects. Wagner (Wagner et al. 2013) showed that SFN pretreatment had a protection on DSS-induced colitis by reducing the expression of inflammatory biomarkers in intestinal mucosa and increasing the expression of Nrf2 dependent genes. Morever, Hubbard (Hubbard et al. 2017) suggested that the broccoli riched in SFN affect the structure of intestinal microbial community and attenuate colitis by alylhydrogen receptor (AHR) dependent mode in Ahr^b/b^ mice. At present, there is no study on the effect of SFN on the intestinal flora of UC mice. Therefore, the purpose of this study is to find out the effect of SFN on the intestinal microbial community of UC mice.

DSS-induced UC in mice is one of the most mature methods at present. The influencing factors of DSS modeling include: molecular weight and concentration of DSS, research environment, mouse species, administration time, etc. (Kitajima et al. 2000). After 7 days of DSS intervention, the mice in the DSS group lost weight, shortened the length of blood stool and colon, and significantly increased the DAI score. The DAI score, colon length and histopathological score in the SFN group were all improved (P < 0.05) than that in the DSS group. The MPO activity of the SFN group was significantly lower than that of the DSS group (P < 0.05), which indirectly proved that SFN reduced the inflammatory degree of acute ulcerative colitis in mice.

Dysbacteriosis of intestinal flora is intimately correlated to the pathogenesis of DSS-induced UC (Chen et al. 2016). The results showed that, compared with the blank group, after 7 days of DSS intervention, the Shannon index of intestinal microflora in other two groups descended, that is, the diversity of microflora decreased. PCoA analysis showed that the distance between the blank group and the DSS group was far from each other, indicating that there was a great difference in the composition of the flora between the two groups, which was same with the previous conclusion of Yang et al. (2017). The DSS intervention could change the β diversity of the intestinal microbial community in mice, that is, change the species composition.

According to the results of phylum level, the three major phyla were *Firmicutes*, *Proteobacteria* and *Bacteroidetes* in the three groups. In comparision to the blank group, the proportion of *Firmicutes* and *Proteobacteria* in the DSS group grown. Meanwhile, the proportion of *Bacteroidetes* and *Verrucomibia* dropped, and the ratio of *F/B* grown (P < 0.05). In comparison to the DSS group, SFN group had lower *Firmicutes*, higher *Bacteroides* and lower *F/B* (P < 0.05). There was no obviously difference in *F/B* between SFN group and blank group. Wu et al. (2016) showed that during AOM/DSS induced colitis related colon cancer in mice, the level of *Bacteroides* decreased and that of *Firmicutes* increased, which was consistent with the results of this experiment. However, Yeom et al. (2016) showed that after DSS intervention, the proportion of *Bacteroides* in mouse intestinal microflora increased, while that of *Firmicutes* decreased. Some studies have suggested that the increase of *F/B* ratio, namely, the increase of *Firmicutes*, and the decrease of *Bacteroides* have a protective effect on inflammatory bowel disease. There are two reasons for the difference: First, the intestinal flora of mice can be affected by many factors such as feed, drinking water, environment and so on. At the same time, the structure of intestinal flora is intimately correlated to the disease factors such as the course of ulcerative colitis, the location of lesions, the degree of inflammation and so on. Secondly, in this study, the number of mice in each group is less, and the individual differences are larger. Therefore, the detection of intestinal flora is also biased.

According to the results of family level, after DSS modeling, in comparision to the blank group, the bacterial of DSS-induced mice changed dramatically. However, the intestinal flora of SFN group was closer to that of blank group. In comparison to the DSS group, *Erysipelototrichaeae* and *Campylobacteraceae* was found significantly decreased in the blank and SFN group. While the relative abundance of *Bacteroidales_S24*-*7*, *Rikenellaceae* and *Prevotellaceae* increased (P < 0.05). According to relevant research reports, *Bacteroidales_S24*-*7* is a kind of intestinal probiotics, which is negatively related to intestinal inflammation. *Rikenellaceae* is a hydrogen producing bacterium, which can selectively neutralize cytotoxic reactive oxygen species (ROS) and protect cells from oxidative stress, thus improving the symptoms of inflammatory bowel disease (Rooks et al. 2014). In general, *Bacteroidales_S24*-*7* and *Rikenellaceae* are more likely to have beneficial bacteria and protect the intestine. The role of *Prevotellaceae* is unclear. On the one hand, it has been reported that *Prevotellaceae* is prominent in IBD patients. Wright et al. (2000) suggested that *Prevetelaceae* may damage the function of intestinal mucosal barrier by producing sulfatase which can degrade mucopolysaccharide, which is increased in IBD patients’ intestinal biopsies. *Prevotellaceae* may also lead to the deterioration of chronic intestinal inflammation in IBD mice. On the other hand, Monk et al. (2016) showed that cranberry bean rich diet can reduce the symptoms of ulcerative colitis in mice, and beans can increase the abundance of *Prevetelaceae* and *Bacteroidales_S24*-*7* in mice intestine. De Cruz et al. (2015) proved that *Prevetellaceae* is a kind of intestinal microorganism which has relation to the remission of inflammatory bowel disease. Therefore, the role of *Prevotellaceae* needs further study in the future.

Currently, in antiretroviral treatment of patients infected with human immunodeficiency virus, it has been proved that *Erysipelototrichaeae* is associated with elevated TNF levels and chronic intestinal inflammation (Dinh et al. 2015). Hubbard et al. (2017) study found that eating broccoli can degrade the relative abundance of *Erysipelototrichaeae* in the intestine of colitis mice and alleviated colitis. *Campylobacteraceae* belongs to *Proteobacteria*. Many *Campylobacter* species may be relative to the pathogenesis of IBD. A meta-analysis by Natalia (Castano-Rodriguez et al. 2017) showed that *Campylobacter* (especially *C. showae* and *C. concisus*) raise the chance of IBD. The results of Lefse analysis of mouse intestinal flora showed that *Erysipelototrichaeae* and *Campylobacteraceae* is also a biomarker in the DSS group. The intervention of SFN can reduce the relative abundance of the two bacteria.

In order to further analyze the specific strains, seven specific strains were found out by comparing the intestinal flora of mice in the blank group and the DSS group on the 14th day of the experiment with the metastats method, the specific species were *Ruminiclostridium*, *Alloprevotella*, *Turicibacter*, *Butyricicoccus*, *Lachnospiraceae_UCG*-*006*, *Ruminococcaceae_UCG*-*013* and *Family_XIII_UCG*-*001*(P < 0.05). The relative abundance of *Butyricicoccus* in the DSS group was the lowest among the three groups, while the relative abundance of *Turicabaracter* was the highest. *Turiribcharacter* belongs to *Erysipelototrichaeae*. Previous studies have shown that the abundance of *Tricibacter* in the gastrointestinal tract of colitis mice (DSS induced mice and IL-22 deficient mice) is decreased (Zenewicz et al. 2013; Collins et al. 2014). More recently, it has been introduced that in AOM/DSS induced colitis related colon cancer, the relative abundance of *Turicabaracter* increased (Wu et al. 2016). In the pathogenesis of human ileal pouch inflammation, which previously suffered from UC, *Turicabaracter* is closely related to the well-known pathogenic bacterium *Clostridium perfringens* (Falk et al. 2007). Therefore, it is believed that *Turibacharacter* has two factors affect with the pathogenesis of mammalian immune system and IBD.

*Butyricococcus* is a *Clostridial cluster IV* that produces butyrate, and its number is reduced in feces of patients with ulcerative colitis (Falk et al. 2007). Butyrate, a short chain fatty acid, was generated in the fermentation of dietary fiber in colon. Butyrate is not only the main energy source of colonic cells, but also maintains colonic homeostasis by regulating various cell functions, including proliferation, differentiation, apoptosis and control of intestinal epithelial permeability (Hamer et al. 2008). In addition, butyrate is also an effective anti-inflammatory medium, which can promote the function of epithelial barrier, induce the ability of colon regulatory T cell differentiation, and inhibit the expression of cytokines (Hamer et al. 2008). In this study, RT-PCR was used to present quantitative analysis. The number of *Butyricicoccus* in the SFN group was more than that in the DSS group, but there was no statistical difference between the two groups. But there was a statistical difference of the number of *Butyricoccus* in the blank group, which was much higher than that in the other two groups. According to the results, the relative abundance of microbial species altered by SFN treatment showed the difference of gut bacterial compositions compared to the DSS group. SFN has the protective effect on intestine by *Butyricococcus*. There are many bacteria can produce Butyrate, such as *Clostridium cluster IV* and *XIVa* bacteria. In this study, only one of them was detected, so the effect of other butyrate producing bacteria on intestinal protection could not be completely denied. In the future experiments, the alleviative effect of SFN on colitis in mice can be further explored.

## Data Availability

Data will be made available through publication and SRA database (PRJNA611676).
